# Pectin Microwave Assisted Extraction from Pumpkin Peels: Process Optimization and Chemical-Physical and Rheological Characterization

**DOI:** 10.3390/foods13193157

**Published:** 2024-10-03

**Authors:** Ilaria Frosi, Raffaella Colombo, Raffaele Pugliese, Chiara Milanese, Adele Papetti

**Affiliations:** 1 Department of Drug Sciences, University of Pavia, 27100 Pavia, Italy; ilaria.frosi01@universitadipavia.it (I.F.); raffaella.colombo@unipv.it (R.C.); 2 NeMO Lab, ASST Grande Ospedale Metropolitano Niguarda, 20162 Milan, Italy; raffaele.pugliese@nemolab.it; 3 Department of Chemistry, Physical Chemistry Section & C.S.G.I., University of Pavia, 27100 Pavia, Italy; chiara.milanese@unipv.it

**Keywords:** pumpkin peels pectin, high methoxyl pectin, by-products, emulsifier agent, agrifood waste

## Abstract

Recently, pectin, a versatile polysaccharide with different industrial applications, has gained significant attention as an eco-friendly and functional ingredient. This study investigates pumpkin peels (*Cucurbita maxima* L., Mantua variety) as a novel source of pectin, using a microwave-assisted extraction method with citric acid-acidified water as solvent. The extraction conditions were optimized using a Design of Experiments approach, considering the solvent-to-solid ratio (SSR), pH, temperature, and extraction time. The optimized conditions (94.8 °C, 5 min, pH 1.5, and 46 mL/g SSR) resulted in a pectin yield of 18.05%. A comprehensive characterization of the extracted pectin was performed, including FT-IR spectroscopy, DSC, TGA, rheological properties, and techno-functional assessments such as water holding capacity and fat binding capacity. The results indicated a high degree of esterification (56.19 ± 0.87%), classifying the pumpkin peels (PP) extract as a high methoxyl pectin. PP pectin demonstrated potential as a stabilizer and emulsifying agent, although its high methoxyl content limits its use as a carrier for targeted bioactive delivery. The findings support the viability of using agricultural by-products to obtain valuable polysaccharides, contributing to waste valorization and sustainable industrial practices.

## 1. Introduction

In the last decades, pectin, a natural polysaccharide mainly found in the plant cell walls, has gained significant attention for its versatile applications in food, pharmaceutical, and cosmetic industries. Pectin is composed of α-(1, 4) linked D-galacturonic acid residues and neutral sugars (L-arabinose and D-galactose) and according to EU regulation is considered food grade when the galacturonic acid content is not lower than 65% [1]. The degree of esterification affects the chemical and mechanical properties of pectin, and therefore its ability to form gels, to stabilize emulsions, and to act as a carrier for targeted drug/bioactive delivery applications, making it a valuable component in several formulations [2,3,4]. Generally, low-methoxyl pectin represents a good solution to develop nanosystems with high loading capacity and encapsulation efficiency which are successfully used for delivering bioactive compounds [5,6] because of their high resistance to gastric digestion conditions and better controlled release at intestinal level [4,7,8]. Differently, the high-metoxyl form is more suitable as stabilizing and emulsifier component in gel forming, due to the high number of hydrophobic interactions generated in the system [2,4].

Recently, there has been an increasing interest in both the development of eco-friendly extraction methods and the utilization of agricultural by-products as source of pectin. This last approach not only addresses environmental concerns but also adds value to waste materials [9]. Currently, the peels of mango, eggplant, banana, melon, potato, and tomato have been investigated as potential source of pectin [10,11,12,13,14,15] in place of citrus peels and apple pomace, already commercially used to this aim [16]. Pumpkin peels (*Cucurbita* spp.) represent a promising yet underexplored source of pectin [17]. Previous studies demonstrated the potential of extracting pectin from various fruit and vegetable wastes, highlighting the efficiency and sustainability of such processes. However, a gap in the literature regarding the specific extraction and characterization of pectin from pumpkin peels is evident [18].

The selection of an appropriate sustainable extraction method is essential not only to maximize the pectin extraction yield but also to preserve its physicochemical, rheological, functional, structural, and biological properties. Recently, advanced techniques such as microwave-assisted extraction (MAE), ultrasound-assisted extraction (UAE), enzyme-assisted extraction (EAE), and pressurized water extraction (PWE) have been developed to enhance the extraction yield of these functional macromolecules while minimizing wastewater production. Compared to conventional hot-water extraction, these more recent methods are more environmentally friendly due to their lower solvent and energy consumption and shorter extraction time [19,20,21,22]. The enzymatic assisted approach is another alternative, especially when the targets are the pectin immunomodulatory properties because the obtained amount of arabinose is more conspicuous with respect to conventional hot acid extraction [23,24]. Differently, MAE is particularly promising for extracting polysaccharides from agricultural sources [22].

In this study, we investigated the potential of pumpkin peels as a source of pectin using a green extraction method assisted by microwaves. The method has been optimized through a Design of Experiments (DOE) approach using water acidified with citric acid as solvent (green solvent). Our aims were firstly to optimize the extraction conditions to maximize pectin yield, and then to characterize the extracted pectin and evaluate its techno-functional properties to assess its suitability for different food and/or cosmetic applications.

## 2. Materials and Methods

### 2.1. Chemicals

Ethanol 96% (*v*/*v*), sodium chloride, and sodium hydroxide were supplied by Carlo Erba (Milan Italy). Methanol (ACS reagent, ≥99.8%), lactic acid (purity grade ≥ 90%), citric acid (purity grade ≥ 99.5%), tartaric acid (purity grade ≥ 99.5%), acetic acid (purity grade ≥ 99.5%), sodium azide (ReagentPlus^®®^, ≥99.5%), and phenolphthalein indicator (ACS reagent) were provided by Merck KGaA (Darmstadt, Germany). Pullulan GFC standard kit was purchased from Waters Corporation (Milford, MA, USA). Water was obtained from Millipore Direct-QTM system (Merk-Millipore, Milan, Italy).

### 2.2. Vegetable Wastes

Pumpkin peels (PP) (*Cucurbita maxima* L., Mantua variety) were obtained after consumption of the vegetables kindly provided by a local Lombard organic farm (G.P.C., Pavia, Italy). PP (thickness 3–4 mm) were freeze-dried and ground into powder using a coffee grinder and then passed through a 500 μm sieve screen to obtain a homogeneous powder. Finally, to ensure proper preservation, PP were stored in amber glass bottles at 4 °C until the use.

### 2.3. Pectin Microwave-Assisted Extraction

The microwave-assisted extraction (MAE) process was carried out using a microwave apparatus equipped with a Teflon vessel system (Ethos LEAN, Sorisole, Italy). 500 mg of PP powder was accurately weighed and placed into the extraction vessel. The extraction conditions were determined based on an experimental design (see Section 2.4), considering the influence of four different parameters on the recovery of pectin from PP: solvent to solid ratio (SSR), temperature (T), pH of aqueous solvent, and irradiation time (t). Initially, the microwave power was set to a maximum of 500 W for 5 min to achieve the desired T. Then, an energy level of up to 500 W was maintained to sustain the samples at the extraction T. Following a ventilation period of 10 min, each sample was filtered through two layers of gauze. The resulting extract was then subjected to centrifugation at 25 °C and 5000 rpm for 10 min. The obtained supernatant was mixed with ethanol (2:1, *v*/*v*) and kept overnight at 4 °C. In the next step, the mixture was again centrifuged at 4 °C and 5000 rpm for 20 min. The resulting precipitate was further purified by resuspending it in water and subsequently diluting it in ethanol until a white precipitate has been formed. The precipitate was then collected after a new centrifugation step at 4 °C and 5000 rpm for 20 min and dried in a hot air oven at 40 °C for 16 h. The particle size of the so obtained material was reduced until it passes through a 500 μm sieve screen. The fine powder was then stored in an amber bottle at room temperature.

The extraction yield of pectin (PEY) was expressed as the percentage ratio of the pectin powder dry weight (WP) obtained after drying in oven with respect to the initial weight of the raw materials (WRM) according to the following equation:

PEY (%) = (WP/WRM) × 100(1)

### 2.4. Design of Experiment (DOE) Approach

The DOE approach was applied to optimize the MAE process and maximize the PEY of pectin from PP.

A screening study was performed to test four different aqueous acidic mixtures in fixed extraction conditions to select the most effective green solvent for pectin recovery from PP. Citric acid, tartaric acid, acetic acid, and lactic acid were used to prepared four different aqueous solutions. The extraction conditions were fixed at pH = 2.6, temperature = 80 °C, time = 5 min, solvent to solid ratio (SSR) = 30 mL/g. The resulting extracts were then subjected to centrifugation, precipitation with EtOH and filtration as reported in Section 2.3.

The response surface methodology (RSM) was then used to optimize the extraction conditions. The most efficient acidic mixture according to the previous screening phase was selected, and PEY was considered as the response variable. The relationship among the variables was represented using a second-order polynomial model with coded independent variables (*X*_*j*,*j*_) that followed the below reported equation:

(2) $$Y=\beta_{0}+\sum_{i=1}^{n}\beta_{i}X_{i}+\sum_{i=1}^{n}\beta_{ii}X_{i}^{2}+\sum_{1\leq i\leq j}^{n}\beta_{ij}X_{i}X_{j}+\varepsilon$$

where Y is the predicted response (yield), *n* is the number of variables, *X_i_* and *X_j_* are the independent variables, β_0_ define the fixed response at central point, β*_i_*, β*_ii_* and β*_ij_* are the linear, quadratic, and interaction coefficients, respectively, and ε is a random error. Analysis of variance (ANOVA) was carried out to determine any significance differences (*p* < 0.5) among the applied extraction conditions. The adequacy of the constructed quadratic model was assessed by on the coefficient of determination (R2), adjusted (R2adj), and the prediction error sum of squares (PRESS). A three-level Box-Behnken Design (BBD) was the most used method to optimize extraction conditions. RSM-BBD with four factors including three repetitions in the central point of experimentation generates a total of 27 experiments (Table 1). The considered experimental ranges (−1; +1) were: pH 1.5–3.5, T 45–95 °C, t 5–15 min, SSR 20–50 mL/g.

### 2.5. Degree of Esterification (DE)

The determination of the degree of esterification (DE) of PP pectin was carried out using a titrimetric approach as detailed by Raji et al. [20]. Pectin dried sample (100 mg) was wetted with ethanol (2 mL) and dissolved in 20 mL of deionized water at 40 °C. After 1 h of stirring, the pectin was completely hydrated, and the solution was added of five drops of phenolphthalein indicator. Subsequently, the solution underwent titration with 0.1 M NaOH and the volume of NaOH solution required to induce a color change was recorded as the initial titer (V1). The subsequent step involved the addition of 10 mL of 0.1 M NaOH and a vigorous agitation followed by a 15 min rest period. Then, 10 mL of 0.1 M HCl was added, and agitation continued until the disappearance of the pink color. The solution was finally titrated with 0.1 M NaOH, and the used volume of the NaOH solution was noted as the saponification titer or final titer (V2). The DE was computed using the following formula:

DE (%) = (V2/(V1 + V2)) × 100(3)

where V2 and V1 are expressed in mL.

Three different preparations were obtained, and each experiment was performed in triplicate.

### 2.6. Pectin Molecular Weight Determination

PP pectin average molecular weight (Mw) and polydispersity index (Pi) were assessed using size exclusion chromatography (SEC) with an Agilent 1200 chromatographic system coupled to a G7162A Refractive Index Detector (RID) (Agilent Technologies, Santa Clara, CA, USA). The mobile phase was a 0.1 M NaCl solution (*w*/*v*) containing 0.02% NaN3 (*w*/*v*), which was filtered through a 0.45 μm Nylon membrane before using it (Merck-Millipore, Milan, Italy). For the chromatography setup, an Ultrahydrogel^®^ 2000 column (12 μm, 7.8 mm × 300 mm) and an Ultrahydrogel^®^ 250 column (6 μm, 7.8 mm × 300 mm) (Waters Corporation, Milford, MA, USA) were connected in series and operated with a consistent flow rate of 0.8 mL/min. Both columns and the detector were maintained at 40 °C. Calibration was performed using narrow pullulan standards in the range of 5 × 10^3^–64^2^ × 10^3^ g/mol (Waters Corporation, Milford, MA, USA).

Samples preparation consisted in the dissolution of PP pectin powder or pullulan standards in 0.1 M NaCl to achieve a final concentration of 1 mg/mL. Prior to injection, samples were filtered using a cellulose microporous membrane filter (0.45 µm, Merck-Millipore, Milan, Italy), and an injection volume of 50 μL was used. Three different PP preparations were obtained and each of them was analyzed in triplicate.

### 2.7. Emulsion Stability

The study of the emulsion stability (ES) for the pectin samples was performed in agreement with the methodology outlined by Mendez et al. [2]. Briefly, the pectin sample solutions (1% and 3%, *w*/*v*) were prepared by dissolving the powder in acidic water (pH 3). Oil-in-water (O/W) emulsions were formulated by dispersing pectin aqueous solution in sunflower oil using a high-speed homogenization apparatus (Ultra-turrax, IKA, Mullheim, Germany) at 10,000 rpm for 5 min, testing two different oil concentrations (35% and 60%, *v*/*v*). The prepared emulsions were stored at 4 °C for 1 and 6 days to test their stability and ES was subsequently calculated using the following equation:

ES (%) = (Volume of remaining emulsion layer/Initial volume of emulsion) × 100(4)

Each experiment was performed in triplicate.

### 2.8. Water Holding Capacity (WHC) and Fat Binding Capacity (FBC)

The determination of water and oil (fat) holding capacities of the pectin samples was performed using a method adapted from Panwar et al. [25] with minor adjustments. To outline the procedure, 200 mg of powdered pectin samples were combined with 2 mL of distilled water or sunflower oil, followed by vigorous vortexing for 1 min at room temperature. Subsequently, the solution underwent centrifugation at 4000 rpm for 20 min. The resulting supernatant was removed, and the remaining residue was weighed. The water and oil holding capacities were quantified as the amount of water or oil retained per 1 g of pectin samples. Each experiment was performed in triplicate.

### 2.9. Fourier Transformation Infrared Spectroscopy (FTIR)

The Fourier-transform infrared (FTIR) spectra of pectin were acquired with a resolution of 4 cm^−1^ using a Nicolet iS10 spectrometer (Nicolet, Madison, WI, USA) and employing the KBr disk method in the range of 4000–450 cm^−1^.

### 2.10. Thermal Properties

PP pectin thermal characteristics were analyzed through differential scanning calorimetry (DSC) and thermogravimetric analysis (TGA), adopting the methodology outlined by Panwar et al. [25]. In the DSC assessment, a small quantity (3 mg) of powdered sample was hermetically sealed within an aluminum pan, introduced into the DSCQ2000 instrument (TA Instruments, New Castle, DE, USA) interfaced with a TA5000 data station and heated from 0 to 300 °C at 10 K/min under nitrogen flux (50 mL/min). Three independent measurements were performed on each sample. The temperature accuracy of the instrument is ±0.1 °C, the precision is ±0.01 °C, and the calorimetric reproducibility is ±0.05%. DSC data were analysed by the Universal Analysis software by TA Instruments.

Furthermore, the thermogravimetric analysis was performed using a Q5000 apparatus (TA Instruments, New Castle, DE, USA) interfaced with a TA5000 data station under nitrogen flux (10 mL/min) by heating about 3 mg of pectin sample in a platinum pan from room temperature up to 600 °C (heating rate 10 K/min). TGA data were analyzed by the Universal Analysis software by TA Instruments.

### 2.11. Rheological and Mechanical Properties

The rheological properties of PP samples (PP aqueous solution) were investigated using a Kinexus DSR Rheometer (Netzsch, Selb, Germany) equipped with a parallel-plate geometry (acrylic diameter 20 mm; gap 34 μm). Sample viscosity was measured using a flow step program with increasing shear rates (0.001–1000 s^−1^) to examine their non-Newtonian behavior and their tendency to exhibit shear-thinning. To assess the storage (G’) modulus PP, frequency sweep experiments were conducted as a function of angular frequency (0.1–100 Hz) at a fixed strain of 1%. Each experiment was performed in triplicate, and data were processed using OriginLab software (OriginPro 2024b).

### 2.12. Statistical Analysis

Results were expressed as the mean ± standard deviations (SD) of the measurements obtained from at least three replicated experiments performed in duplicate/triplicate. Differences were considered significant at *p* < 0.05. Statistical analysis was carried out using Microsoft Office 365 and Statgraphics Centurion 19 software (Statgraphics Technologies, Inc., The Plains, VA, USA) was used for experimental design, model fitting, and data analysis.

## 3. Results and Discussion

In the last years, pectin generated great interest as it can be extracted from by-products and/or agricultural wastes, thus meeting the principle of circular economy. In particular, the investigation focused on the possibility to use it as emulsifying and stabilizing agent or as carrier for intestinal targeted formulations [3,18]. At this regard, we investigated pumpkin peels (*Cucurbita maxima* L., Mantua variety) as a possible source of pectin.

### 3.1. Pectin Extraction and Characterization

A green extraction method assisted by microwaves in acidic aqueous medium was optimized using a DOE approach. Four different acids (citric, tartaric, lactic, and acetic acids) were tested in the screening phase in fixed conditions selected according to the literature data, i.e., pH 2.6, 5 min, 80 °C and 1:30 g/mL [9,26,27]. Considering the obtained PEY%, water acidified with citric acid (mother solution 73 g/130 mL) was the most promising solvent (PEY 4.6%) followed by acetic (4.3%), lactic (4.0%), and tartaric acid (2.6%). Therefore, the comprehensive experimental design was planned to use citric acid to acidify water. Table 2 reported the observed pectin extraction yield values and the considered experimental ranges (−1; +1) which were SSR 20–50 mL/g, pH of acidic water 1.5–3.5, T 45–95 °C, and extraction time 5–15 min.

The mathematical formula of the second-order polynomial equation derived from the response surface was as follows:

PEY (%) = −54.8662 + 2.03343 × SSR + 35.8689 × pH + 1.23813 × T − 3.58301 × t − 0.0203867 × SSR2 − 1.15128 × SSR × pH + 0.00295733 × SSR × T + 0.0209667 × SSR × t − 2.04417 × pH2 − 0.8075 × pH × T + 1.6025 × pH × t − 0.00459067 × T2 + 0.00054 × T × t + 0.04825 × t2 + 0.00967778 × SSR2 × pH − 0.0000306667 × SSR2 × T − 0.00028 × SSR2 × t + 0.0763333 × SSR × pH2 + 0.00000773333 × SSR × T2 + 0.0815 × pH2 × temperature − 0.2775 × pH2 × t + 0.00232 × pH × T2(5)

The PEY obtained under the different extraction conditions ranged from 2.48% and 14.12% (Table 2). Our findings generally were in line with those reported by other researchers who previously investigated the extraction of pectin from various waste materials, such as carrot pomace [28], *Citrus medica* peel, and pistachio green hull [19,29] or higher than other fruit’s peel [24]. Notably, ANOVA results highlighted pH as a crucial factor affecting pectin extraction (*p* < 0.05), with a negative impact on yield (Table 3). In fact, we observed a significant increase in PEY at the lowest pH value applied (pH 1.5). This phenomenon was attributed to the hydrolysis of polysaccharides under strong acidic conditions, which enhances the pectin release and solubility, as previously reported for the pectin extracted from pistachio green hull [29] and carrot pomace [28]. Furthermore, we observed a generally positive effect on PEY of the increase in SSR, T, and extraction time, even though their individual impacts were not statistically significant. In literature, several studies reported that PEY increases as T increases and generally the highest PEY were obtained at the highest extraction temperature applied [19,28].

Indeed, we recorded the highest pectin recovery at the highest temperatures (70–95 °C), likely due to enhanced solubility and diffusivity of pectin from plant tissues into the solvent with the increasing of temperature. Regarding SSR, it is well-documented that an increased contact between the material and the solvent, along with a high water diffusivity into cells, may lead to a higher yield, as reported in the extraction of polysaccharides from melon peels by Golbargi et al. [22]. Similarly, in terms of extraction time, it is generally advisable to keep irradiation times below 15 min to avoid decomposition, degradation, and polysaccharides hydrolysis, which can reduce the yield [22].

The results obtained in our experiments demonstrated a strong agreement with predicted values, as evidenced by the high coefficients of determination (R2: 96.71%; R2adj: 78.59%).

**Figure 1:**
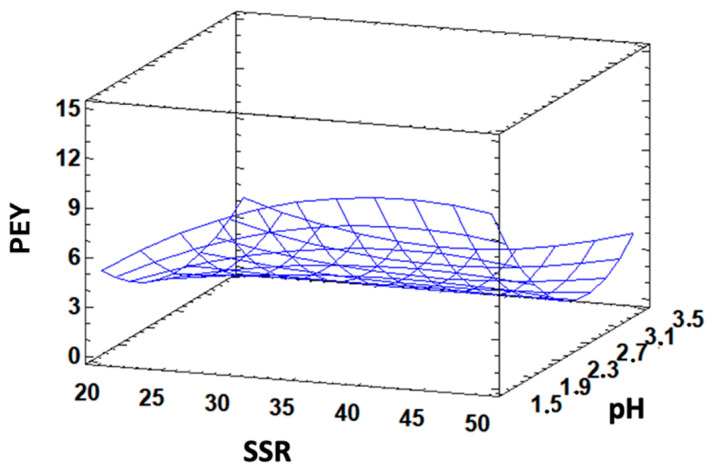
Response surface plot showing the effect of SSR and pH on pectin yield (PEY) at 70 °C and 10 min extraction time.

Considering the surface plots obtained for PEY as a function of SSR and pH (Figure 1), it is evident that low pH and high SSR provided the highest yield in fixed time and temperature conditions. pH is effectively the significant variable that drives the highest yield, differently from variation in SSR which does not provide significant changes in PEY. Consequently, the optimal conditions for maximizing PEY were determined to be as follows: 94.8 °C, 5 min, pH 1.5, and SSR of 46 mL/g. Under these conditions, the calculated PEY was 18.05%, with a 95% confidence interval for the mean value ranging from 11.14% to 24.96%. The results obtained by analyses of three additional experiments performed under these optimal predicted conditions, indicated that the mean of PEY was 22.8 ± 1.94%, further confirming the accuracy of our fitted model. By applying these optimized experimental extraction conditions, we obtained higher yields than other researchers using the same extraction method (MAE), but different solvent (generally simply water), for pumpkin peel (11.3 ± 0.2%) [30], grape pomace (11.23%) [31], and Sentul peel (Sandorium koetjape) (12.66%) [32]. Differently, our PEY values were similar to those obtained using MAE in combination with citric acid for pistachio gene hull (22.1 ± 0.5%) [29] and lime peel (19.63 ± 0.78%) [33], thus supporting the validity of our results.

### 3.2. Pectin Chemical Characterization

The obtained polysaccharide was then characterized to investigate its chemical composition.

**Figure 2:**
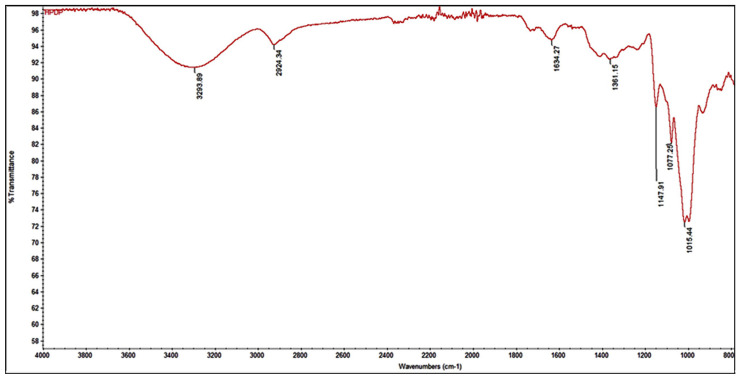
FT-IR spectrum of isolate pectin polysaccharide from pumpkin peels.

Figure 2 reports the FT-IR spectrum of the polysaccharide, showing the typical bands of PP pectin already reported in literature [17,34], and so confirming the pectin identity.

In fact, the band in the range of 3200–3600 cm^−1^ corresponds to the vibration of both the intra- and inter-hydrogen bonds of OH groups within the pectin molecule. This vibration was particularly strong at 3294 cm^−1^. Another notable peak at 2924 cm^−1^ can be attributed to the stretching of the C-H bonds in the methyl groups present on the sugar rings of the molecule structure. Furthermore, two distinct absorption bands between 1770 cm^−1^ and 1600 cm^−1^ indicated the presence of C=O groups, specifically methyl-esterified (COO-R) and ionic carboxyl (COO-) groups. It is important to note that the relative intensity of the ester band increased according to the DE of the pectin (a higher DE corresponds to a stronger intensity in the ester band), while the intensity of the carboxylic stretching band decreases [17]. The 1200–1800 cm^−1^ range, known as the fingerprint region, reveals distinct bands characteristics for PP pectin. Notably, the typical peaks at 1077 cm^−1^ and 1015 cm^−1^ were correlated with the vibration of neutral arabinose and galactose-based glycans within the pectin molecule [17].

**Figure 3:**
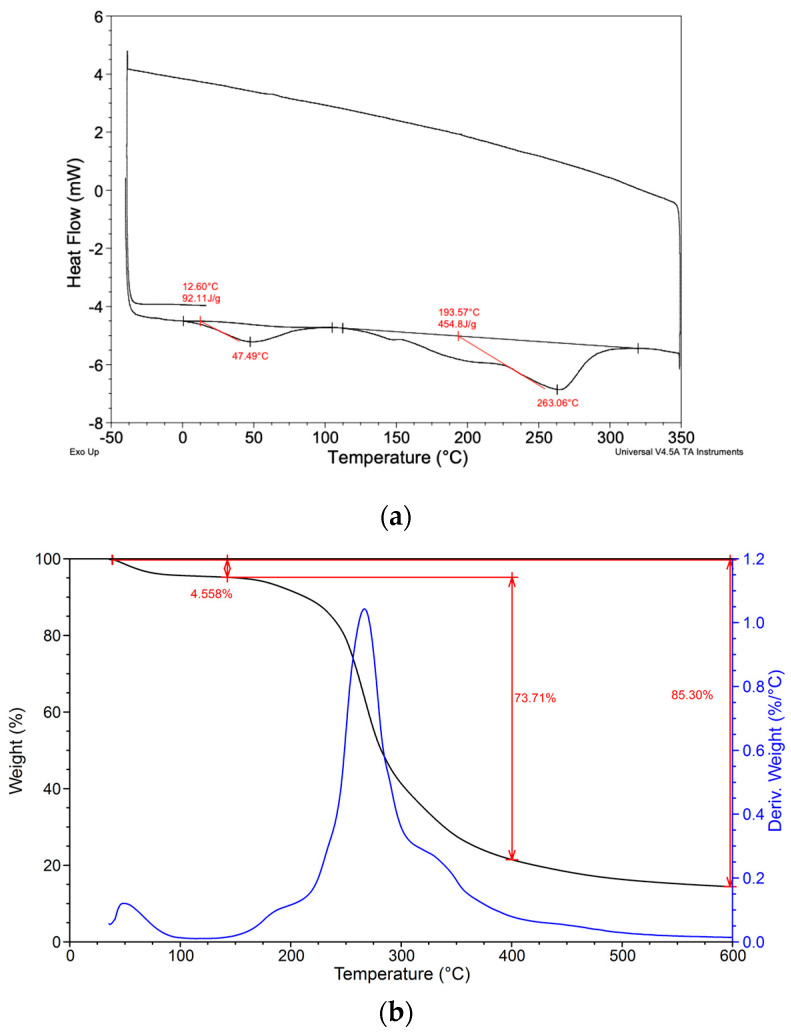
DSC (**a**) and TGA (**b**) analyses of the extracted pumpkin peels pectin.

Additionally, thermal analysis using DSC (Figure 3a) supported the presence of physically bound water, which is released at temperatures below 100 °C, and revealed multiple decomposition processes occurring up to 263.06 °C. TGA data confirmed a weight loss of 4.6%, corresponding to the removal of free water from the sample (Figure 3b). A more significant loss of water was observed between 200 °C and 350 °C, indicative of the pyrolytic decomposition of the pectin chain and the cleavage of hydrolytic bonds. These processes continued until the complete decomposition of organic substances at 600 °C, leaving behind a carbon-based inorganic skeleton. These findings aligned with the trends observed in the thermal performance of an ultrasound assisted extracted pectin (UAEP) from citrus peels by Panwar et al., highlighting a greater thermal stability of UAEP from food wastes compared to the commercial citrus pectin [25].

**Figure 4:**
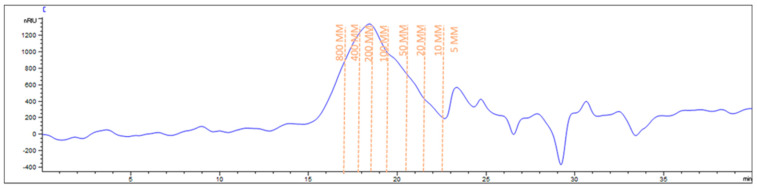
Molar weight (Mw) distribution of pumpkin peel pectin (1 mg/mL in NaCl 0.1 M) by SEC-RID system.

The Mw and the DE highly affect the application field of pectin. Therefore, these parameters were investigated to gain more insight on the chemical features of the isolated polysaccharide. SEC was used to investigate the average Mw and Mw distribution of PP pectin, after the construction of a calibration curve with pullulans (using the external standard method). The molecular parameters were extrapolated by the chromatogram registered for PP pectin (Figure 4) which highlighted the presence of a heterogeneous population of polymers possessing a number average molecular weight (Mn) of 131.33 ± 3.53 kDa and an average Mw of 351.55 ± 31.40 kDa, higher than that observed for pumpkin peel pectin isolated by other researchers [17,35]. The dispersity index (PDI) calculated as the ration Mw/Mn was of 2.67 ± 0.18, a value which closely resembled that of the commercial citrus pectin (PDI = 2.0 ± 0.0), which can be attributed to the high purity of pectin [35]. The Mw distribution was quite narrow, thus confirming the results obtained by Mendez et al. [35] for watermelon rinds pectin obtained under extraction conditions very similar to those applied for PP pectin (i.e., high-temperature (95 °C) and low-pH (about 1)), even if PP pectin Mw was higher (watermelon rinds pectin Mw 106 ± 2.69 kDa). Conversely, Torkova et al. [17] reported lower values ranging from 72.2 ± 7.7 kDa to 169.1 ± 16.0 kDa while Salima et al. [34] found 171.25 ± 39.26 kDa. Remarkably, our PP pectin exhibited a Mw value similar to that reported by Salima et al. [34] for pumpkin pulp (407.48 ± 8.45 kDa), as well as for some commercial citrus pectin (389.3 ± 9.1 kDa) and sugar beet pulp pectin (590.3 ± 11 kDa) [36]. Indeed, to the best of our knowledge, pectin extracted from Mantua pumpkin peels has never been investigated before this work.

As regards the DE, PP pectin exhibited a DE value greater than 50% (DE = 56.19 ± 0.87%), classifying it as a high methoxyl pectin. This result was in line with those previously reported for microwave-assisted extraction of pumpkin pectin (DE = 55–63%) [30,37]. This classification involved a content of hydrophobic groups higher than that of hydrophilic groups [35]. Considering this feature, PP pectin was not suitable for the use as a potential carrier agent, as low methoxyl pectins (LMP) were employed for this purpose [18]. In fact, LMP are characterized by the abundance of free carboxyl groups on the polysaccharide chains, which can interact with cations like Ca^2+^ and Zn^2+^ to form rigid gel structures that are well-suited for active entrapment [38]. Therefore, PP pectin features indicated a more promising use as a stabilizer or emulsifying agent.

### 3.3. Pectin Structural and Techno-Functional Properties

**Figure 5:**
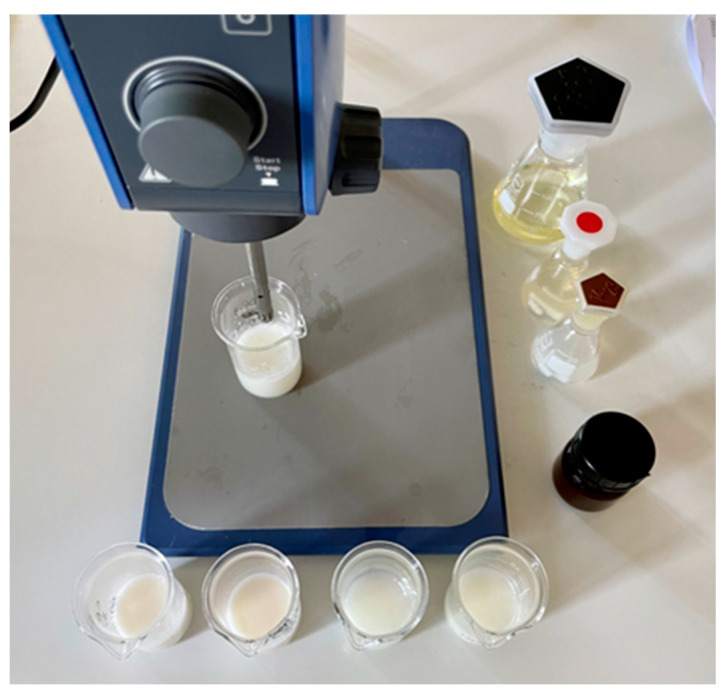
Image of different emulsions prepared by using various concentration of pectin (1–3% *w*/*v*) and sunflower oil (35–60% *w*/*v*).

Considering the tecno-functional properties, WHC and FBC were examined to better understand the interaction between the hydrophobic and lipophilic components of pectin and the number of hydrophilic/lipophilic groups on its surface. A high WHC is associated to a better suitability as texturizer, while a high FBC is related to its use as an emulsifier and stabilizing agent. WHC referred to the amount of water held per gram of pectin, while FBC referred to the amount of oil bound per gram of pectin. The WHC of PP pectin was 1.55 ± 0.25 g water/g, similar to the value reported for melon peels pectin (1.88 g water/g) [22], but lower than that of citrus peels pectin (3.07 ± 0.09 g water/g) [25]. Conversely, the FBC was 2.51 ± 0.19 g oil/g, similar to the values observed for citrus peels pectin (2.37 ± 0.05 g oil/g) and pistachio green hull (2.02 ± 0.38 g oil/g) [11]. Therefore, PP pectin exhibited a proportion of hydrophobic region higher than lipophilic ones, consistent with the trend observed in melon peels pectin by Golbargi et al. [22]. This causes the pectin polymer’s increased ability to stabilize O/W emulsions. To better investigate this property, different emulsions were prepared using different pectin concentrations (1% and 3%, *w*/*v*) and different concentration of sunflower oil (35% and 60%, *w*/*v*) (Figure 5) and their stability was monitored during 7 days at 4 °C.

Interestingly, as shown in Table 4, the stability of the different emulsions did not significantly change at 4 °C. In particular, the emulsions with the highest oil content (E2 and E3) exhibited the greatest stability, independently from the concentration of pectin. When the water content was higher compared to the oil one, separation of the oil phase may be observed on the surface of the emulsion. These findings were in line with those reported by Mendez et al. for pectin extracted from watermelon rinds (a vegetable belonging to the same botanical family), which was more effective as emulsion stabilizing agent when the oil content was the highest [39].

**Figure 6:**
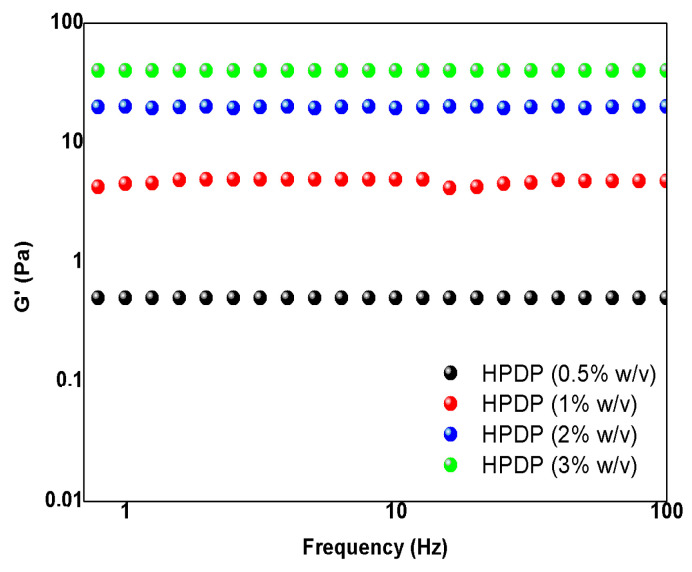
Rheological studies on pumpkin peel pectin: frequency-dependent oscillatory rheology (0.1–100 Hz) to test the mechanical properties.

Preliminary rheological studies were also performed on PP pectin to investigate the viscoelastic properties. Three different concentrations (0.5, 1, 2, and 3%, *w*/*v*) of pectin aqueous solution were tested by using shear-rate rheology. In Figure 6 the mechanical properties of pectin solution are reported. The storage modulus (G’) represented the stiffness, and it increased by increasing pectin concentration (from 0.5 to 60 Pa). This behavior aligned with the typical patterns observed for soft hydrogel matrices and it can be attributed to the formation of entanglements between pectin chains at the highest concentrations, resulting in enhanced material stiffness. Consequently, these properties could be linked to the concentration of pectin in the solution. In fact, as noted in the rheological studies, the G’ increased with the concentration of pectin, indicating the formation of a stronger network. This suggests that, at higher concentrations, the pectin molecules form entanglements or interactions that lead to a gel-like behavior, where the G’ becomes independent of frequency. These entanglements could be the result of hydrophobic interactions and hydrogen bonding between the pectin chains, which are especially prominent in high methoxyl pectins like the one described in this study.

**Figure 7:**
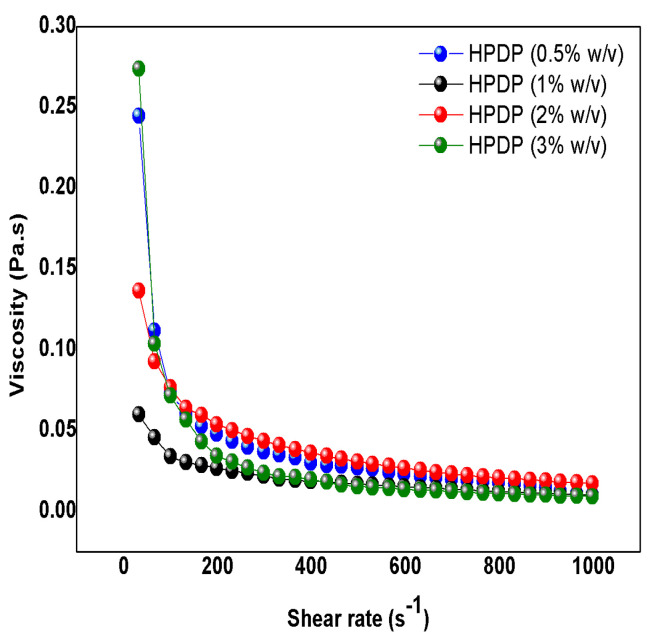
Rheological studies on pumpkin peel pectin: flow curves to test the viscosity.

At the tested concentrations, pectin can form a sufficiently robust gel network that maintains a certain level of elasticity, as evidenced by the complete independence of the storage modulus. Simultaneously, the viscosity may decrease under applied stress due to the shear thinning effect. This interplay between elasticity and flow behavior highlights the complexity of pectin-based materials. While they exhibit significant elasticity, their flow behavior is sensitive to applied stresses, making them versatile for various applications. We believe this duality is a key characteristic that enhances the utility of pectin in different contexts, both as a stabilizer and a thickening agent. When the flow curves of apparent viscosity were analyzed (Figure 7), a non-Newtonian, shear-thinning, and pseudoplastic response was observed, indicating that the viscosity decreased as the applied shear rate increased. This phenomenon was previously documented in pectin extracted from citrus peels using ultrasound techniques and it is the result of the development of a robust pectin network occurring at high concentrations which leads to increased pectin collision rates and, subsequently, to a decrease in viscosity [25]. This viscosity profile underscored the potential use of pectin as both stabilizer and thickener agent, with potential applications not only in food products but also in injectable formulations [25].

## 4. Conclusions

This study aimed to explore the feasibility of using pumpkin peels (*Cucurbita maxima* L., Mantua variety) as a source of pectin, particularly for its potential as a carrier agent for intestinal targeted formulations or as an emulsifier and stabilizing agent in gel formation. Using a green extraction method assisted by microwaves and optimized through a DOE approach, we successfully extracted pectin using water acidified with citric acid. The extraction process was comprehensively analyzed, revealing that pH is a crucial parameter affecting pectin extraction yield, with lower pH values significantly enhancing the extraction yield due to the hydrolysis of polysaccharides under strong acidic conditions. Our findings demonstrated that a pectin yield higher than those reported in similar studies involving different waste materials was achieved under optimized conditions (94.8 °C, 5 min, pH 1.5, and an SSR of 46 mL/g, PEY 22.8 ± 1.94%), thereby validating the efficiency of our extraction process.

The characterization of the extracted pectin confirmed its chemical identity (FT-IR), and its thermal behaviour was consistent with those of pectin extracted by other food wastes, indicating good thermal stability. Also the techno-functional properties, including WHC and FBC, were comparable to those of pectin from other sources, suggesting its potential use as a stabilizer or emulsifying agent. Preliminary rheological studies revealed that the pectin exhibited non-Newtonian, shear-thinning behavior, which is desirable for applications as a thickener and stabilizer. Furthermore, the MW and DE suggested a high methoxyl pectin classification with a DE of 56.19 ± 0.87. Therefore, this classification indicated PP pectin unsuitable for the use as a carrier agent for intestinal targeted delivery due to the lack of free carboxyl groups necessary for gel formation with cations. However, the thermal stability and techno-functional properties make it a valuable stabilizer or emulsifying ingredient in food and pharmaceutical industries.

In conclusion, this research contributes to the sustainable use of agricultural by-products and provides insights into the potential applications of pumpkin peels pectin in different food sectors. Additionally, understanding the impact of extraction parameters on pectin yield and properties will help in developing efficient and eco-friendly extraction processes, paving the way for future innovations in the utilization of vegetables-based polysaccharides.

Future studies could explore modifications to the extraction process to produce LMP from pumpkin peels, thereby expanding its applicability in targeted drug delivery systems.

## Figures and Tables

**Table 1:** Box-Behnken Design with three factors for extraction of pectin from pumpkin peel. Coded variables.

Run (No.)	SSR	pH	T	t
1	−1	−1	0	0
2	+1	−1	0	0
3	−1	+1	0	0
4	+1	+1	0	0
5	0	0	−1	−1
6	0	0	+1	−1
7	0	0	−1	+1
8	0	0	+1	+1
9	−1	0	0	−1
10	+1	0	0	−1
11	−1	0	0	+1
12	+1	0	0	+1
13	0	−1	−1	0
14	0	+1	−1	0
15	0	−1	+1	0
16	0	+1	+1	0
17	−1	0	−1	0
18	+1	0	−1	0
19	−1	0	+1	0
20	+1	0	+1	0
21	0	−1	0	−1
22	0	+1	0	−1
23	0	−1	0	+1
24	0	+1	0	+1
25	0	0	0	0
26	0	0	0	0
27	0	0	0	0

**Table 2:** Box-Behnken Design for MAE water acidified extraction of pectin from pumpkin peel. Actual variables. Extraction yields (PEY%) are also reported.

Run (No.)	SSR (mL/g)	pH	T (°C)	t (min)	PEY (%)
1	20	1.5	70	10	4.30
2	50	1.5	70	10	9.66
3	20	3.5	70	10	3.68
4	50	3.5	70	10	3.42
5	35	2.5	45	5	3.59
6	35	2.5	95	5	3.82
7	35	2.5	45	15	4.28
8	35	2.5	95	15	4.78
9	20	2.5	70	5	3.59
10	50	2.5	70	5	3.6
11	20	2.5	70	15	3.58
12	50	2.5	70	15	4.00
13	35	1.5	45	10	5.59
14	35	3.5	45	10	4.42
15	35	1.5	95	10	13.79
16	35	3.5	95	10	5.10
17	20	2.5	45	10	4.58
18	50	2.5	45	10	3.52
19	20	2.5	95	10	3.18
20	50	2.5	95	10	4.96
21	35	1.5	70	5	14.12
22	35	3.5	70	5	4.14
23	35	1.5	70	15	10.02
24	35	3.5	70	15	4.34
25	35	2.5	70	10	3.06
26	35	2.5	70	10	2.48
27	35	2.5	70	10	2.58

**Table 3:** The analysis of variance of the Box-Behnken model for pectin extraction yield for MAE-water acidified process.

Source	Sum of Squares	df	Mean Square	F Ratio	*p* Value
A:SSR	0.05	1	0.05	0.02	0.8872
B:pH	61.31	1	61.31	30.26	0.0053
C:T	0.13	1	0.13	0.07	0.8103
C:t	0.68	1	0.68	0.34	0.5933
AA	0.35	1	0.35	0.17	0.6989
AB	7.65	1	7.65	3.77	0.1240
AC	2.02	1	2.02	1.00	0.3749
AD	0.04	1	0.04	0.02	0.8924
BB	67.50	1	67.50	33.32	0.0045
BC	14.14	1	14.14	6.98	0.0575
BD	4.62	1	4.62	2.28	0.2054
CC	4.56	1	4.56	2.25	0.2078
CD	0.02	1	0.02	0.01	0.9290
DD	7.76	1	7.76	3.83	0.1220
AAB	9.48	1	9.48	4.68	0.0965
AAC	0.06	1	0.06	0.03	0.8722
AAD	0.20	1	0.20	0.10	0.7699
ABB	2.62	1	2.62	1.29	0.3188
ACC	0.01	1	0.01	0.01	0.9460
BBC	8.30	1	8.30	4.10	0.1129
BBD	3.85	1	3.85	1.90	0.2401
BCC	4.21	1	4.21	2.08	0.2231
Total error	8.10	4	2.03		

**Table 4:** Emulsion stability (ES) percentage after 1 and 7 days at 4 °C of the 4 different emulsions prepared with PP pectin.

Emulsion No.	Composition	Emulsion Stability (ES %)	
		Day 1	Day 7
1	PP 1%—oil 35%	60.0 ± 0.1	58.3 ± 0.1
2	PP 1%—oil 60%	90.0 ± 3.3	91.7 ± 2.3
3	PP 3%—oil 35%	58.3 ± 2.4	56.7 ± 2.3
4	PP 3%—oil 60%	98.3 ± 2.4	98.3 ± 2.3

## Data Availability

The original contributions presented in the study are included in the article material, further inquiries can be directed to the corresponding author.
